# Entropy in Heart Rate Dynamics Reflects How HRV-Biofeedback Training Improves Neurovisceral Complexity during Stress-Cognition Interactions

**DOI:** 10.3390/e22030317

**Published:** 2020-03-11

**Authors:** Veronique Deschodt-Arsac, Estelle Blons, Pierre Gilfriche, Beatrice Spiluttini, Laurent M. Arsac

**Affiliations:** 1 Univ. Bordeaux, CNRS, Laboratoire IMS, UMR 5218, 33400 Talence, France; estelle.blons@u-bordeaux.fr (E.B.); pierre.gilfriche@u-bordeaux.fr (P.G.); laurent.arsac@u-bordeaux.fr (L.M.A.); 2 CATIE-Centre Aquitain des Technologies de l’Information et Electroniques, 33400 Talence, France; 3 URGOTECH, 15 avenue d’Iéna, 75116 Paris, France; bspiluttini@urgotech.fr

**Keywords:** refined composite multiscale entropy, complexity, central autonomic network, heart rate variability, interconnectivity

## Abstract

Despite considerable appeal, the growing appreciation of biosignals complexity reflects that system complexity needs additional support. A dynamically coordinated network of neurovisceral integration has been described that links prefrontal-subcortical inhibitory circuits to vagally-mediated heart rate variability. Chronic stress is known to alter network interactions by impairing amygdala functional connectivity. HRV-biofeedback training can counteract stress defects. We hypothesized the great value of an entropy-based approach of beat-to-beat biosignals to illustrate how HRVB training restores neurovisceral complexity, which should be reflected in signal complexity. In thirteen moderately-stressed participants, we obtained vagal tone markers and psychological indexes (state anxiety, cognitive workload, and Perceived Stress Scale) before and after five-weeks of daily HRVB training, at rest and during stressful cognitive tasking. Refined Composite Multiscale Entropy (RCMSE) was computed over short time scales as a marker of signal complexity. Heightened vagal tone at rest and during stressful tasking illustrates training benefits in the brain-to-heart circuitry. The entropy index reached the highest significance levels in both variance and ROC curves analyses. Restored vagal activity at rest correlated with gain in entropy. We conclude that HRVB training is efficient in restoring healthy neurovisceral complexity and stress defense, which is reflected in HRV signal complexity. The very mechanisms that are involved in system complexity remain to be elucidated, despite abundant literature existing on the role played by amygdala in brain interconnections.

## 1. Introduction

Although it has become increasingly evident that physiological systems are complex, in the sense that many interdependent components interact at different hierarchical levels and simultaneously operate at different time scales, there can be no direct quantification of complexity in living systems. Rather, an intuitive approach with considerable appeal has been that physiological/biomedical signals that are generated by such systems may carry information on the system complexity, its self-organization, and potential adaptability, so pointing to signal complexity analysis is a reliable way to examine coordinated interactions in neurophysiological networks. Prior knowledge of system organization might allow for anticipating, to some extent, system responses through a dynamical organization as well as long-term (persistent) adaptations. Accordingly, in controlled conditions, a logical expected system behavior should help in strengthening the link between system complexity if one can demonstrate that signal complexity change concurrently [1]. Ultimately, changes in output signal complexity should reflect interconnectivity at neurophysiological levels [2,3,4].

It has been known for years that the brain and the heart exhibit permanent top-down and bottom-up interactions that are critical beyond cardiovascular health, for behavioral, cognitive, and emotion regulations [5]. As a link between these two organs, a flexible network of neural structures has been extensively described, which is dynamically organized in response to a variety of internal and external stimuli. This complex circuitry is nicely embodied in the conceptual model of neurovisceral integration [6,7,8], in which prefrontal-subcortical inhibitory circuits that are critically involved in self-regulation are linked with the heart via the vagus nerve [5,6,7,8,9,10,11]. The overall functioning of the many constitutive hierarchical components and their multiple interactions have been studied so far through quantifying high-frequency (HF) modulations in heart rate variability, which is a marker of vagal tone in the system signal output. The so-called vagally-mediated heart rate variability (HF-HRV) has shown a critical non-invasive transdiagnostic marker of psychological states [12] because of the inhibitory action of the prefrontal cortex (PFC), which shapes cognitive-behavioral responses [6,8,13,14].

Studies encompassing psychology, cardiovascular, and neuroimaging domains provide converging evidence of a link between short-range (HF) HRV dynamics and the prefrontal subcortical circuits through an intricate network [15,16,17,18]. They collectively point to the critical role of network functional activity for cognitive and emotional self-regulation [5]. Additionally, they designate amygdala as a critical target of stress/anxiety in this circuitry, playing a critical role in system interconnectivity. As a clear illustration of amygdala-dependent interconnectivity, statistical maps of structural covariance in neuroimaging confirmed that amygdala interconnections encompass wide portions of cortical and subcortical regions, which serves as a crucial node in intricate circuits [19]. Amygdala functional connectivity is necessary for a dynamic coordination within the central autonomic network (CAN). Stress-induced disruption in amygdala-driven interconnectivity is clearly reflected in the HRV output signals [18,19].

It follows from above that, reasonably enough, one might expect a causal link between amygdala functional connectivity, a coordinated neurovisceral integration, and complexity in the healthy unconstrained CAN.

Researchers have recently more closely associated mood and cognition to complexity markers in HRV dynamics [20,21,22]. In agreement with the above assumption, the main observation is that complexity in heartbeat dynamics grows with brain activity, but vanished with stress. Further, multiscale entropy in HRV has been suggested as a reliable marker of coordinated neurovisceral integration during stress-cognition interactions [23], although this is a new recent hypothesis that should be addressed further, by manipulating e.g*.,* stress management. While the response to stress in humans is a healthy adaptive function in situations of acute challenge, a prolonged exposure to stressors might cause persistent dysregulations [12], which affects the CAN, as reflected in a systematically blunted vagal tone [24,25]. Heightened resting vagal HRV helped in demonstrating that the functioning of the whole network can be restored, which has promoted the design of specific interventions that are able to enhance the vagal traffic in people with corrupted cortico-subcortical inhibition [26,27,28]. Among such interventions, HRV biofeedback (HRVB) training has been shown to be an easy-to-use and reliable method that restores cortical inhibitory control [27], which is beneficial in chronic stressed subjects [29]. The HRVB technique consists in slowing the spontaneous respiratory rate that drives vagal activity toward the same natural frequency of the sympathetic cardiovascular control, to around around 0.1 Hz [26], which establishes resonance among vagal and sympathetic baroreflex control loops. Reaching so-called cardiac coherence provides an increased baroreflex gain, which improves the vagal afferent traffic and bottom-up brain stimulations and, ultimately, restores a degraded psychophysiological state [30,31], or improves defense against episodic stressing events, as shown in students during examinations [32,33]. To date, we have no idea how the signal complexity might change with HRVB training.

The aim of the present study was to provide a novel application of a complexity-based method to evaluate coordination in a neurophysiological network, the CAN, through complexity in its output signal, HRV dynamics.

For that, Refined Composite Multiscale Entropy (RCMSE) in heartbeat time-series was assessed during stress-cognition interactions in self-reported moderately stressed participants, before and after HRVB training to trigger system adaptations. We hypothesized that improved stress defense is associated with greater signal complexity, which could reflect better neurovisceral coordination.

## 2. Materials and Methods

### 2.1. Participants

The procedures are in agreement with the French law that allows for performing experiments on humans and publishing the obtained results without requiring approbation and ID by an IRB or ethical committee, because the experiments are part of the research training that has been approved by the faculty steering committee, which has full responsibility on the training program. The experimental group (‘Heart Rate Variability Biofeedback’: HRVB group) consisted of 13 healthy participants (eight males and five females, aged 42.5 ± 15.1 years) performing administrative work at the faculty. They all reported being somewhat stressed (see stress quantification below) and they have difficulty for balancing work, family, and lifestyle. Six unstressed people (four males and two females of similar age) served as the control group (CTRL group).

None of the participants were receiving medical treatment before enrollment. They were required to abstain from food or drink for two hours before the HRVB training procedure, scheduled on early morning and early evening before breakfast and lunch. The participants abstained from caffeine ingestion on the experimental days. After five-weeks of HRVB training, three participants of the HRVB group dropped-out of follow up. They argued for too high constraints being linked to the day-to-day HRVB training. Thus, the final sample undergoing both assessments encompassed 10 subjects (seven males and three females).

### 2.2. Experimental Protocol

The experimental protocol consisted of two 10 min sequences that were separated by a few minutes that were dedicated to fill psychological questionnaires. The same sequences were repeated before and after HRV biofeedback training. During each sequence, the subjects stayed quietly seated in front of a computer, breathing at spontaneous rate, while the heartbeat time series were recorded, as described below. The resting conditions corresponded to the first 10 min of watching a calm and soothing documentary. During the second 10 min sequence, the participants performed cognitive tasks in a controlled stressful environment. They had to respond to 31 questions that were displayed on a computer screen, which needed the mental processing of logic, memorization, and calculation in a balanced proportion. Questions were created with the E-Prime software (Psychology Software Tools Inc., Pittsburgh, PA, USA), so that the participants answered by pushing dedicated keys on a keyboard. The added stressors had the form of predetermined response time, visual feedbacks for false responses, and an attentive and evaluative audience (two people standing near the participant and taking notes). Flashing lights crowd noises, car honks, and sirens completed the set of added stressors. The number of questions, the type of logic memorization and mental calculation questions, the negative feedback, and the two people for evaluative audience were different before and after HRVB training to avoid undesired consequences of habituation.

### 2.3. Heart Rate Variability Biofeedback (HRVB) Training Procedure

HRVB training was assigned to the experimental group for five weeks. The participants had to control their breathing rate at ~ 6 cycles per min without changing their natural tidal volume, in quiet conditions for 5-min periods twice a day (morning and evening). A connected device that was developed by URGOTECH linked by Bluetooth to a smartphone application, URGOfeel, guided the controlled breathing rate^®^ (URGOTECH, Paris, France). As feedback, heart rate was detected non-invasively by infrared finger photoplethysmograph and processed to detect respiratory sinus arrhythmia (RSA) and the presence of a unique mode (frequency) in HRV, which characterizes cardiac coherence, thereby, suitable conditions for afferent cortical-subcortical stimulation through vagal afferent traffic.

### 2.4. Psychological Tests

The participants filled out a series of questionnaires. The Spielberger’s State-Trait Anxiety Inventory (STAI [34]) consists of 20 items that measured the subjective feelings of apprehension, tension, nervousness, and worry. The NASA Task Load Index (NASA-TLX [35]) was developed to assess cognitive workload. The participants were asked to evaluate six components on a scale: mental demand, physical demand, temporal demand, performance, effort, and frustration level, as well as the weight of each component, allowing for the calculation of a global cognitive workload index. The Perceived Stress Scale (PSS [36]) wherein 14 items provided information on the frequency of thoughts and feeling regarding the encountered situation.

### 2.5. Heart Rate Recordings and Analyses

Cardiac interbeat intervals (R-to-R peaks interval durations) were recorded while using a Polar H10 chest belt that was linked by Bluetooth to a smartphone. Polar chest belts demonstrated great accuracy in assessing RR intervals when compared to ECG recordings [37,38]. The RR (intervals) time series were exported to Matlab (Matlab 2016b, Matworks, Natick, MA, USA) and then analyzed for heart rate variability (HRV) using custom-designed algorithms. Raw data were inspected for artifacts; occasional ectopic beats (irregularity of the heart rhythm involving extra or skipped heartbeats, e.g., premature ventricular contraction and consecutive compensatory pause) were visually identified and manually replaced with interpolated values from adjacent RR intervals. The root mean square of the differences between successive intervals (RMSSD) was computed in the time-domain because RMSSD is an index of very short-term variability that dominantly reflects short-latency vagal modulations [39]. Power Spectral Density (PSD) was obtained by using a Fourier transform after cubic spline resampling of the RR signals (4 Hz). Prior to the computation of discrete Fourier transform (DFT, without windowing), 4 Hz-resampled series were detrended by using a detrending method based on the smoothness priors approach [40]. The smoothing parameter was adjusted at 500 which corresponds to the way a time-varying FIR (finite impulse response) high pass filter with a cut-off frequency around 0.033 Hz operates.

Spectral power was computed in the low frequency band (LF-power; 0.04–0.15 Hz) and the high frequency band (HF-HRV; 0.15–0.4 Hz), and then interpreted as pure sympathetic and dominantly vagal activities, respectively. LF power/HF power was computed as an indicator of sympatho-vagal balance.

**Figure 1:**
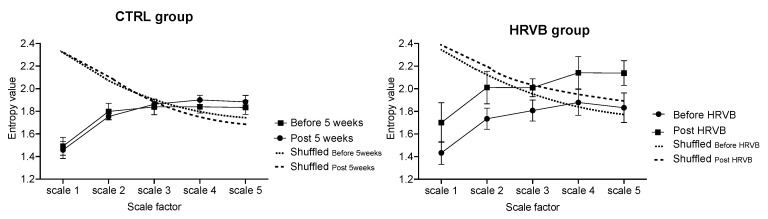
Refined composite multiscale entropy (RCMSE) analysis of RR interval time series. Sample entropy values at time scales 1 to 5 during stressful cognitive tasking are reported. The RCMSE curves for the surrogate shuffled time series are also presented. The entropy index represents the trapezoid approximation of the area under each curve: (**left**) Unchanged values in the control group; (**right**) Higher entropy after heart rate variability biofeedback (HRVB) training.

Complexity in the RR time series was captured by computing Refined Composite Multiscale Entropy (RCMSE), an improved method for obtaining sample entropy [41,42] at several time scales from coarse-grained time series [43] of moderate length [44]. The rationale of using multiscale entropy analysis lies in the fact that complexity in neurophysiological networks provides them with the essential capacity to operate over many timescales, which makes the rate of information staying high and quite steady over a range of scales, in strong contrast with systems shifting towards disorder (white noise) or strict order (mode locking) [45]. Here, the overall degree of complexity of HRV signals was calculated by integrating the values of sample entropy that were obtained over the shortest scales, which corresponds to the above described vagal control of heart rate. Refined composite multiscale entropy (RCMSE) improved the accuracy of MSE by reducing the probability of inducing undefined entropy, which is especially useful when analyzing the short time series of cardiovascular dynamics [23], as recently shown [44]. Detailed methods for computing MSE and RCMSE can be found, respectively, in [45] and [44,46]. The RCMSE curve is obtained by plotting sample entropy values for each coarse-grained time series as a function of scales. The cardiac entropy index is the area under the corresponding RCMSE curves (areas calculated using the trapezoidal rule) Figure 1 [44,45]. The entropy indices were computed after pre-processing time series using empirical mode decomposition (EMD) [47], as recommended by Gow et al. [48]. EMD decomposes a signal into a sum of intrinsic mode functions (IMFs) and a residual trend. This residual trend was subtracted to remove the drift, in order to avoid error in entropy assessments [48].

### 2.6. Statistical Analyses

The quantitative variables were expressed as mean, standard deviation (SD), and coefficient of variation (CV %).

The normality of each dataset was determined using the d’Agostino–Pearson normality test. One-way analyses of variance followed by paired or unpaired *t-*tests with Bonferroni corrections for multiple comparisons were applied to observe the effects of HRVB training on the psychological and physiological indices. The effect Size with Hedge’s *g* was calculated. Values above 0.80 were adopted with high magnitude (‘large’), above 0.5 with medium (‘med.’) and 0.2 with small (‘small’) magnitude. The Pearson’s correlation coefficient was computed for analyzing the relationship between two variables. The Receiver Operating Characteristic (ROC) curve through Sensitivity, Specificity, Area Under Curve defined the efficacy of the HRV indices in time (RMSSD), frequency (HF-HRV, LF-HRV; LF/HF ratio), and non-linear (entropy) domains. The respective *p* values were used between the pre- and post-HRVB training set by Youden Index.

All of the statistical calculations were performed using GraphPad (Prism 8, version 8.2.1, 2019) and XLSTAT (Addinsoft, 2019, XLSTAT statistical and data analysis solution, Long Island, NY, USA).

## 3. Results

### 3.1. Psychological Markers

**Figure 2:**
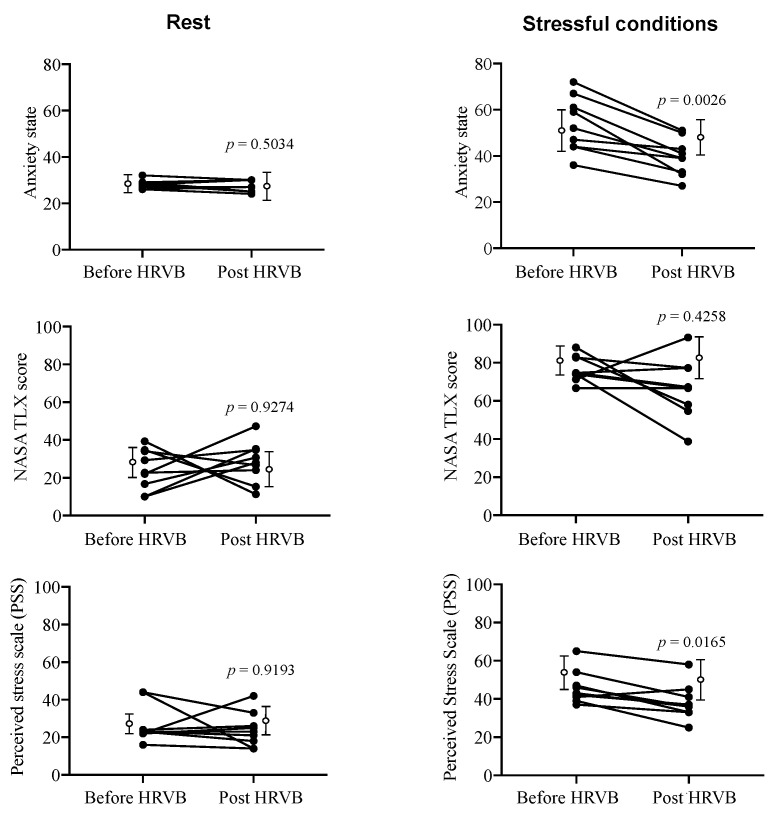
Individual changes in psychological markers induced by five-weeks HRV biofeedback training (HRVB) (filled circles) at rest (**left**) and during stressful cognitive tasking (**right**). Open circles indicate mean and standard deviation obtained in control group and illustrate the absence of changes.

Figure 2 illustrates the main adaptations that were induced by HRVB training as regards psychological markers. The adaptations were exclusively observed during the stressful cognitive condition, not at rest Figure 2. State anxiety and Perceived Stress were significantly lower after HRVB training (*p* = 0.0026 and *p* = 0.0165, respectively), whereas the perceived cognitive load (NASA TLX score) remained unchanged (*p* = 0.4258). This observation is not trivial, because it supports the idea that a lower stress/anxiety is not the consequence of less attention being paid to the cognitive task (since the cognitive load is intact), but a pure HRVB training beneficial effect on anxiety and perceived stress when facing our stressful controlled conditions. The absence of changes in the participants of the control group confirmed the pure effect of training. Overall, psychological markers indicate that HRVB training helped participants to prevent a rise in anxiety/stress while facing the stressful cognitive task.

### 3.2. HRV-Based Autonomic Markers

**Figure 3:**
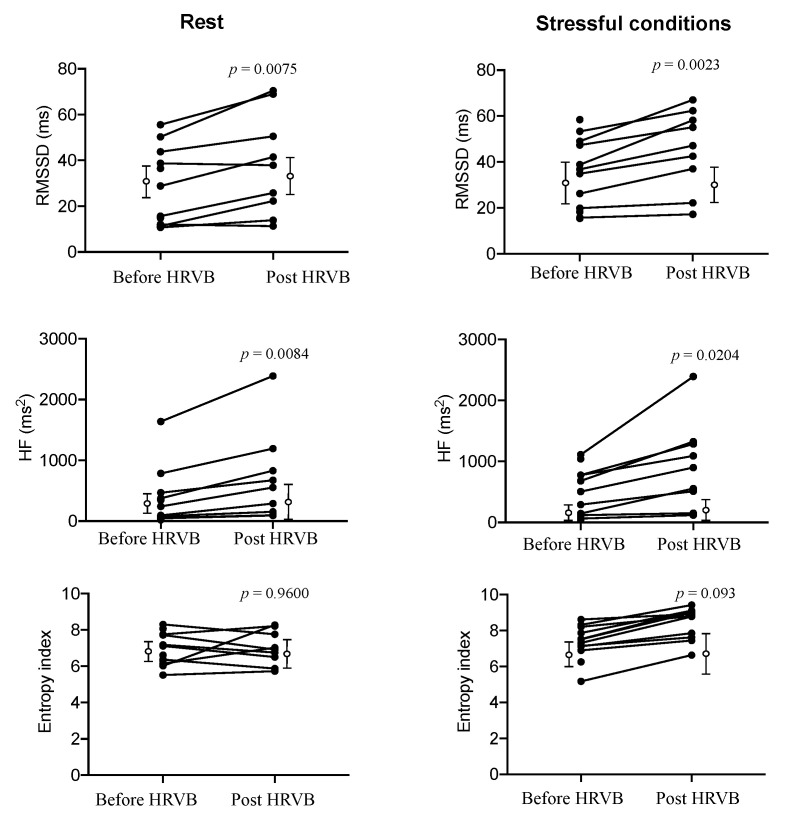
Individual changes in RMSSD, HF-HRV, and Entropy index markers induced by five-weeks HRV biofeedback training (HRVB) (filled circles) at rest (**left**) and during stressful cognitive tasking (**right)**. Open circles indicate mean and standard deviation obtained in control group.

The main effects of HRVB training on HRV at rest and during stressful cognitive conditions are indicated in Table 1 and in Figure 3 where averaged values in the control group are indicated in order to highlight the specific training effect in the experimental group, not seen in the control group Figure 3.

The RMSSD and HF-HRV values indicate a small effect size of training on vagal activity at rest and a moderate effect during stressful cognitive tasking. The sensitivity analysis demonstrated that the main effects of HRVB training were effective during stressful cognitive tasking Table 2.

**Figure 4:**
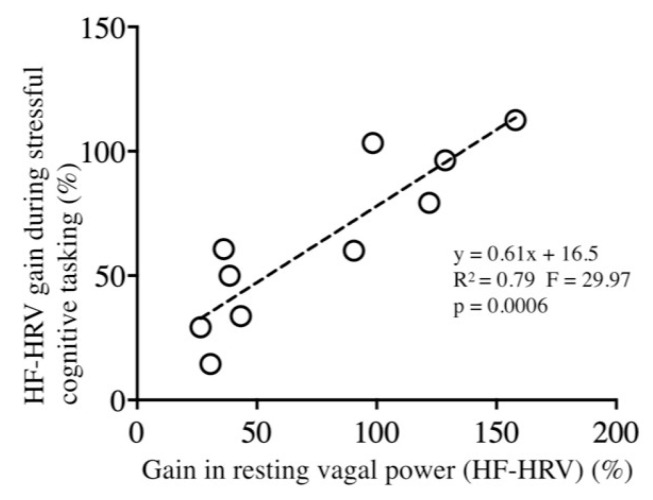
Correlation analysis between HRVB training gain (calculated as post–pre)/pre * 100) in high-frequencies (HF)-power during stressful cognitive tasking vs. rest.

We highlighted a link between training benefits at rest and those that were observed during stressful cognitive tasking Figure 4; those participants with the most important gain in resting HF-HRV (resting vagal tone) correlatively demonstrated the most important gain in HF-HRV during stressful cognitive tasking (R^2^ = 0.789, F = 29.97, *p* = 0.0006, Figure 4).

Taken together, the above adaptations in vagal activity after training indicate that enhanced vagal tone at rest could help in reaching higher vagal control during a stressful task.

Remarkably, autonomic adaptations to training were more consistent and clear-cut when assessed with a complexity marker, RCMSE. First, entropy exhibited a small coefficient of variation (~20%), which contrasts with CV in other markers (mostly >>40%, Table 1). More clearly as well, the entropy index signed benefits of HRVB training, reaching the highest value of effect size during stressful tasking Table 1, as well as higher statistical performances in sensitivity analysis Table 2.

**Figure 5:**
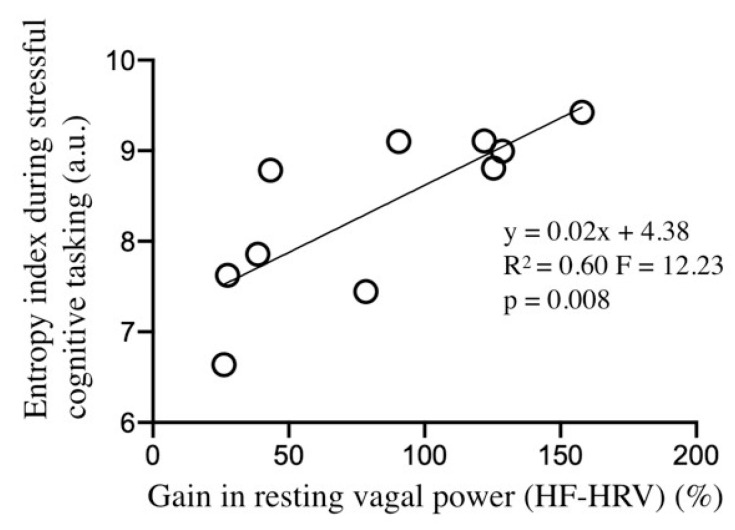
Correlation analysis between post-training entropy index during stressful cognitive tasking vs. training-induced gain in HF-power (calculated as post–pre)/pre * 100).

Finally, a link was observed between individual gain in resting vagal power and entropy; with those participants with greater improvement in resting vagal control reaching a higher level of entropy during stressful cognitive tasking (R^2^ = 0.59, F = 11.42, *p* = 0.009, Figure 5).

## 4. Discussions

The main aim of the present study was to show the value of a complexity-based analysis, refined multiscale entropy (RCMSE), to identify changes in the coordinated interconnectivity of the central autonomic network (CAN). It was hypothesized that a coherent profile in entropy changes during stress-cognition interactions provides a meaningful approach of CAN complexity and neurovisceral adaptability to HRVB training. The main finding in this sense was that entropy in the output signal was heightened despite stress, thanks to HRVB training. This was accompanied with training benefits on vagal activity, which is known to prevent disruption in amygdala functional connectivity [13,18]. We suggest that our results collectively represent a coherent basis to gain improved knowledge on neurovisceral coordination, and by so doing illustrate the link that one can make between system complexity and signal complexity. Here, psychological, vagal, and complexity responses to HRVB training offer a coherent vision of neurovisceral complexity and may open new perspectives for HRV-complexity approaches of heart-brain interactions.

To obtain a realistic interpretation of a link between system complexity and signal complexity in our conditions, a pre-requisite is that autonomic responses and long-term adaptations match with previous observations that consistently report on the link between vagal activity, anxiety, and interconnectivity in the neurovisceral circuitry when the brain has to respond emotionally and cognitively. A low resting vagal HRV and/or an excessive vagal tone withdrawal when one faces an acute challenge has been associated with poor health and poor effectiveness in coping with a variety of stimuli and challenges [49]. These defects in vagal autonomic activity are generally associated with cortico-subcortical dysfunctions [50], which lead to highly susceptibility to amygdala disconnection and a corrupted behavioral and cognitive flexibility. Prolonged exposure to stress is one obvious candidate at the origin of such dysfunctions, being reflected in impaired heart vagal control. In agreement, our moderately stressed participants demonstrated low vagal resting activity and, more critically, a blunted vagal response during stressful tasking Table 1 before HRVB training; remarkably, the vagal activity rose after daily HRVB training thanks to repeated bottom-up vagal stimulations of the brain, especially during stressful cognitive tasking [31,51]. Previous studies have shown that HRV biofeedback training has the capacity to enhance inhibitory control [52] and improve overall self-regulation, autonomic stability, and psychosocial well-being [31], which can be explained by true persistent CAN adaptations. The present work brings about additional support for effective neurovisceral remodeling, being illustrated by measurable benefits of HRVB training that extended beyond resting conditions, in vagally-mediated responses to stressful cognitive tasking, which illustrates profound changes that can be mobilized under different conditions. The correlation between gain in resting and stressful cognitive tasking gain in vagal activity highlights the extended ability to mobilize new resources thanks to the improved CAN dynamic organization Figure 4.

The capacity to maintain high vagal activity at rest as well as during a cognitive task is critical in stress defense [32], and it has been shown to be a pre-requisite for preserving cortico-subcortical inhibition, thereby amygdala functional connectivity [5]. Hence, as a first and critical step for building up a complexity-based concept of neurovisceral coordination, it should be acknowledged that our participants demonstrated an improved vagally-mediated ability to preserve amygdala functional activity during stressful cognitive tasking thanks to HRVB training. This was illustrated here by better vagal (HF-HRV) activity and sympatho-vagal (LF/HF) balance concomitant with a reduction of perceived stress and anxiety after training, which contrasts with poorer status before HRVB training reflected in those markers.

Interestingly enough, we show a correlation between the training-induced gain in vagal activity, which confers better psychophysiological status to a participant [12], and the entropy index that is associated to the stressful cognitive task Figure 5. Hence, better signal entropy while stressful tasking is not without connection with the facilitated vagal control, notwithstanding the fact that entropy demonstrated greater sensitivity than most other autonomic markers to discriminate the training effects Table 1 and Table 2. Our interpretative hypothesis, although speculative at this stage, is that the ability of the entropy index to consistently reflect training induced improvements in neurovisceral integration in the presence of stress might have roots in preserved activity of the main target of anxiety, the amygdala functional connectivity. The reason why entropy, which is a complexity marker, demonstrated that great value might lie in the fact that amygdala activity is critical for subsystems-interconnectivity, as shown by neuroimaging [19]. We speculate here on a possible link between neurovisceral complexity and amygdala functional connectivity, given the multiple connections within and between large portions of cortical (e.g., prefrontal, cingulate, and insula) and subcortical (e.g., striatum, hippocampus, and midbrain) regions and vagal pathways, with the amygdala as a central node in this connected network [19,53]. Giving credit to this overview of the CAN dynamic organization shows the high potential of complexity-based approaches to decipher functional connectivity and coordination in a neurophysiological network.

While we use RCMSE here, a large panel of complexity-based methods for analyzing interbeat time series can be drawn. To evoke a few representative examples, sample entropy has been applied to wavelet-based decomposition in very-low (VLF), low (LF), and high-frequencies (HF) at different ages [54]; multiscale entropy has been applied to diurnal vs. nocturnal series at different ages and health status [45]; the monofractal scaling exponent has been shown to change with ageing, cardiac health, and disease [55,56]; multifractality disruption has been evidenced in heart failure [57]; and, more recently, multifractility-multiscale analysis of both cardiac and vascular dynamics provided a deeper understanding of sexual dymorphism in autonomic control of heart and peripheral vascular districts [58]. In each case, the added value of obtaining complexity metrics was highlighted. The present study is in the same vein by attempting to associate RCMSE with CAN complexity.

Using a multiscale entropy approach for that is not without limitations. Mainly, the significance of sample entropy at given scale strongly depends on the length of the analyzed time series [45,46]. We illustrate the great adequacy of RCMSE, a complexity-based method purposely developed for shorter series [44] to highlight system complexity by showing consistent sample entropy estimates in the present approach from scale 1 to scale 5 Figure 1, from 500–600 data samples series.

In brief, here we suggest that a complexity-based approach of cardiac interbeat time series during stress-cognition interactions is helpful in understanding complexity changes in an intricate central-autonomic neurovisceral circuitry. This statement finds strong support in the combined markers of cognitive load, state anxiety, perceived stress, vagal activity, and entropy, which collectively offered a coherent vision of cooperative mechanisms. Although advanced knowledge on the role of amygdala has recently been provided, an obvious limitation in the present study is the absence of any metrics regarding amygdala functional connectivity or direct evidence of changes in brain networks complexity. Therefore, we conclude that, although HRV biofeedback training appears to be an effective means to preserve a healthy complexity, and that this very property is reflected in HRV entropy, the very mechanisms that link neurovisceral coordination to signal complexity remain to be established.

## Figures and Tables

**Table 1:** Mean, standard deviations (SD) and coefficient of variations (CV %) of time-, frequency-, and nonlinear markers extracted from Heart Rate Variability during rest and stressful experimental conditions before and after 5-weeks HRVB training.

Markers	Before HRVB			Post HRVB				
	Mean	SD	CV (%)	Mean	SD	CV (%)	Effect size	*p* Value
RMSSD (ms)								
rest	27.4	16.9	61.6	38.0	22.0	57.8	−0.541 small	0.007
stress	34.5	15.4	44.4	45.4	17.4	38.4	−0.662 med.	0.002
LF-HRV (ms^2^)								
rest	824	653	79.3	1161	647	55.8	−0.418 small	0.230
stress	1268	957	75.5	1070	732	68.4	0.232 small	0.925
HF-HRV (ms^2^)								
rest	352	465	132.2	697	736	105.6	−0.560 small	0.008
stress	472	394	83.3	925	709	76.7	−0.790 med.	0.020
LF/HF								
rest	3.23	1.39	43.0	2.51	1.29	51.4	0.084 small	0.050
stress	3.08	2.24	72.8	1.60	1.21	75.6	0.732 med.	0.021
Entropy index								
rest	6.86	0.29	4.23	7.00	0.32	4.57	−0.478 small	0.889
stress	7.33	0.94	12.90	8.43	0.89	10.53	−1.198 large	0.003

RMSSD: Root Mean Square of the Successive Differences; LF-HRV: Low Frequency; HF-HRV: High Frequency; LF/HF: ratio between Low and High Frequencies; Entropy: entropy index calculated from RCMSE analysis.

**Table 2:** Efficacy of HRV indices in time-, frequency-, and nonlinear domains in the discrimination of HRVB training effects at rest and during stressful cognitive tasking.

Variables	Sensitivity	Specificity	Youden Index	AUC	*p* Value
RMSSD (ms)					
rest	0.589	0.567	0.156	0.648	0.255
Stress-task	0.617	0.588	0.204	0.694	0.135
LF-HRV (ms^2^)					
rest	0.594	0.571	0.165	0.657	0.227
stress-task	0.528	0.521	0.049	0.546	0.722
HF-HRV (ms^2^)					
rest	0.713	0.674	0.318	0.722	0.088
stress-task	0.708	0.684	0.392	0.731	0.075
LF/HF					
rest	0.611	0.583	0.194	0.685	0.155
stress-task	0.774	0.785	0.560	0.824	0.013
Entropy index					
rest	0.704	0.644	0.349	0.793	0.097
stress-task	0.813	0.799	0.612	0.818	0.010

RMSSD: Root Mean Square of the Successive Differences; LF-HRV: Low Frequency; HF-HRV: High Frequency; LF/HF: ratio between Low and High Frequencies; Entropy: entropy index calculated from RCMSE analysis; AUC: area under the ROC curve.
